# Foray movements are common and vary with natal habitat for a highly mobile bird

**DOI:** 10.1002/ece3.11096

**Published:** 2024-02-29

**Authors:** Caroline L. Poli, Kenneth D. Meyer, Philip C. Darby, Sarah J. Dudek, Gina Kent, Robert J. Fletcher

**Affiliations:** ^1^ Department of Wildlife Ecology and Conservation University of Florida Gainesville Florida USA; ^2^ Avian Research and Conservation Institute Gainesville Florida USA; ^3^ Department of Biology University of West Florida Pensacola Florida USA

**Keywords:** apparent survival, dispersal, emigration, exploration, hydrology, individual movement, natal habitat, wetland

## Abstract

Understanding dispersal is central to interpreting the effects of climate change, habitat loss and habitat fragmentation, and species invasions. Prior to dispersal, animals may gather information about the surrounding landscape via forays, or systematic, short‐duration looping movements away from and back to the original location. Despite theory emphasizing that forays can be beneficial for dispersing organisms and that such behaviors are predicted to be common, relatively little is known about forays in wild populations. Theory predicts that individuals that use forays may delay dispersal and such behaviors should increase survival, yet empirical tests of these predictions remain scarce. We tested these predictions in a natural system using the critically endangered snail kite (*Rostrhaumus sociabilis*), a wetland‐dependent raptor. We GPS tracked 104 snail kites from fledging through emigration from the natal site across their breeding range to understand the demographic consequences of movement. We found that forays were common (82.7% of individuals tracked), and natal habitat played an important role in the initiation, execution, and outcome of foray behavior. The effect of foraying on survival was indirect, where forayers emigrated later than non‐forayers, and individuals that emigrated later had the highest survival. Poor hydrological conditions in the natal environment were especially important for eliciting forays. Finally, females responded more strongly to natal hydrology than males, making more forays and significantly longer, more distant trips. These results emphasize the fundamental role of natal habitat for determining behavioral patterns, strengthen links between individual movement decisions and their demographic consequences, and provide an important behavioral focal point for interpreting movement tracks that would not otherwise be captured by conventional movement models.

## Graphical abstract

Despite theory predicting the benefits of forays for dispersal, there is relatively little empirical evidence of forays and even less on their fitness consequences. We address this gap using an extensive GPS‐tracking dataset from snail kites tracked from fledging through emigration from the natal site, a critical life stage when many animals are vulnerable to mortality. We find that natal habitat is related to the timing, distance, and duration of forays and that foraying kites initiate dispersal later than kites who do not foray, with indirect survival benefits.

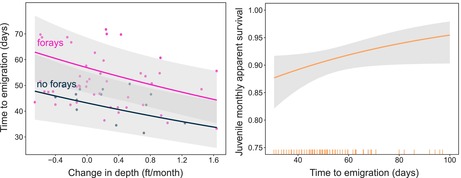

## 1 INTRODUCTION

Natal dispersal (hereafter “dispersal”), the movement of an animal from its natal site to its first breeding location, is an essential process that influences gene flow and is central to interpreting the effects of climate change, habitat loss and fragmentation, and species invasions (Clobert et al., 2012). Dispersal has three components: departure (emigration), transience and settlement (Bowler & Benton, 2005; Clobert et al., 2009). Prior to departure, animals often use exploratory movements to gain information about their environment, which can increase dispersal success and survival (Clobert et al., 2009; Zollner & Lima, 1999). One important type of exploratory behavior is the “foray.” Forays are systematic, semi‐recursive, short‐duration movements with the same origin and destination that provide individuals with information about the surrounding landscape (Conradt et al., 2003). Tracking technology has revealed the potential for foray movements across unprecedented scales, and such behaviors may occur in several taxa (Barve et al., 2020; Jayatilaka et al., 2014; Selonen & Hanski, 2010). Yet forays are likely under documented and remain poorly understood.

Foraying is predicted to influence the outcome of dispersal, but there is conflicting information on whether forayers experience net benefits or costs. On the one hand, models suggest that forays can improve dispersal success of young animals and guide adult movement patterns (Conradt et al., 2003). Another possible benefit is that foraying may lead to delayed dispersal (Fattebert et al., 2019), which can increase survival and future breeding site recruitment (Jungwirth et al., 2015; Tarwater & Brawn, 2010). Forayers may invest more time and energy gathering information about new sites while still benefiting from access to food and shelter within the home habitat (Reed et al., 1999), whereas non‐forayers may invest less time and effort exploring but incur risk and expend energy finding food and shelter in an unfamiliar site. On the other hand, lack of familiarity with habitat during movement could lead to increased mortality rates for forayers (Avril et al., 2012). Foraying is favored in heterogenous landscapes, and should, therefore, be common in many natural systems (Conradt et al., 2003), yet information tends to come from cooperative species that may have different motivations for movement than non‐cooperative species. Studies that compare the demographic consequences of foraying versus non‐foraying strategies in wild populations are rare (for examples see Kingma et al., 2016; Walters et al., 1992).

For young animals that have not yet dispersed, natal habitat may play a major role in shaping foray behavior (Clobert et al., 2009) and determining individual fate. Yet the role of natal habitat is likely to vary with different aspects of foraying for at least three reasons (Table 1). First, low‐quality natal habitat may lead to higher movement rates (Janin et al., 2012) and earlier movement (Fattebert et al., 2019). Poor environmental conditions may, therefore, increase the likelihood of forays, lead to earlier initiation of forays, and increase the number of forays since animals from poor habitat should not want to return. Second, the natal site may influence the distance and duration of forays if animals assess the quality of nearby sites based on conditions in the home patch; however, natal effects may be weak because of other factors (Clobert et al., 2009). For instance, sex‐based differences in movement are common and might arise during forays because of differences in body size and locomotion ability (Clay et al., 2020), or, if foray and dispersal distance are linked, as a consequence of sex‐biased dispersal strategies (Clobert et al., 2009). Third, foray experience and habitat quality in the natal site concurrently should drive the timing of emigration. Delayed emigration has been observed in studies where animals are provided supplemental food (Fattebert et al., 2019), suggesting that poor‐quality natal habitat can result in earlier emigration (Robles et al., 2022). Detailed information on foray behavior can advance understanding of the dispersal process, especially during the often‐cryptic juvenile life stage.

**TABLE 1:** Expectations for movement behavior during different stages of exploration in response to natal habitat and individual sex.

Stage	Movement behavior	Natal habitat effect (poor quality)	Sex‐based differences
Initiation of forays	Probability of foray	Higher foray probability	Unlikely
Initiation of forays	Time to first foray	Shorter time to first foray	Unlikely
During forays	Number of forays	Weak effect; more forays	♀ Likely more forays
During forays	Distance traveled in forays	Weak effect; less travel	♀ Likely more travel
During forays	Time spent in forays	Weak effect; shorter travel time	♀ Likely longer travel time
After forays	Time to emigration	Shorter time to emigration	Unlikely

We investigated the role of natal habitat on movement behavior in the snail kite (*Rostrhaumus sociabilis*), a wetland‐dependent raptor that is endangered in the United States. Snail kites feed almost entirely on aquatic apple snails (*Pomacea* sp.) and occupy a patchy and unpredictable ecosystem consisting of discrete wetland units spread over central and south Florida, USA (Reichert et al., 2020). First‐year survival is lower than that of adults (Reichert et al., 2016), and movements during the first 4 months after fledging are especially risky in terms of increased likelihood of mortality (Bennetts & Kitchens, 1999). After fledging, young kites often remain in the natal wetland for ~30–90 days. During that time, kites may make exploratory movements (Bennetts & Kitchens, 2000), leading to questions about whether natal habitat could influence individual movement behavior prior to dispersal, and explain, at least in part, variation in first‐year survival rates. Concerning conservation of snail kites in the US, attention has been paid to managing habitat to increase reproduction, especially via wetland hydrology (Fletcher et al., 2021; USFWS, 1999). However, hydrologic targets that benefit post‐fledging birds are likely to differ from targets for nesting. There is potential to manage habitat during the post‐fledging stage, and understanding fine‐scale movements can clarify the relevance of the natal site for survival.

We predict that hydrology and snail density in the natal wetland during the time of fledging will influence movement behavior of fledgling snail kites before, during, and after forays. Adult kites from the same cohort adopt similar between‐wetland movement strategies (Valle et al., 2017), which potentially points to natal habitat as a driving factor for movement (Fletcher et al., 2015; Poli et al., 2022). Prior research also has shown that adult snail kites move in response extremely low and high water levels (Bowling et al., 2012; Martin et al., 2006), and both high and low food availability (Bennetts & Kitchens, 2000); therefore, we expect unusually wet and dry conditions in natal habitat to prompt fledglings to move earlier, farther, and longer. We also expect other factors, such as an individual's sex and experience with habitat outside of the natal wetland to play a major role in the distance and duration of forays. For instance, in any year, adult females are 28% more likely to disperse than adult males (Reichert et al., 2016). We also predict that forayers will emigrate later from the natal site compared to non‐forayers and that this difference in timing will lead to higher survival of forayers compared to non‐forayers. Ultimately, understanding the causes and consequences of variation in movement patterns of post‐fledging snail kites will help clarify the extent to which existing theory regarding forays plays out in real‐world scenarios and provide management guidelines that can be implemented at the natal site to benefit this critically endangered species.

## 2 MATERIALS AND METHODS

### 2.1 Study area and tagging

**FIGURE 1:**
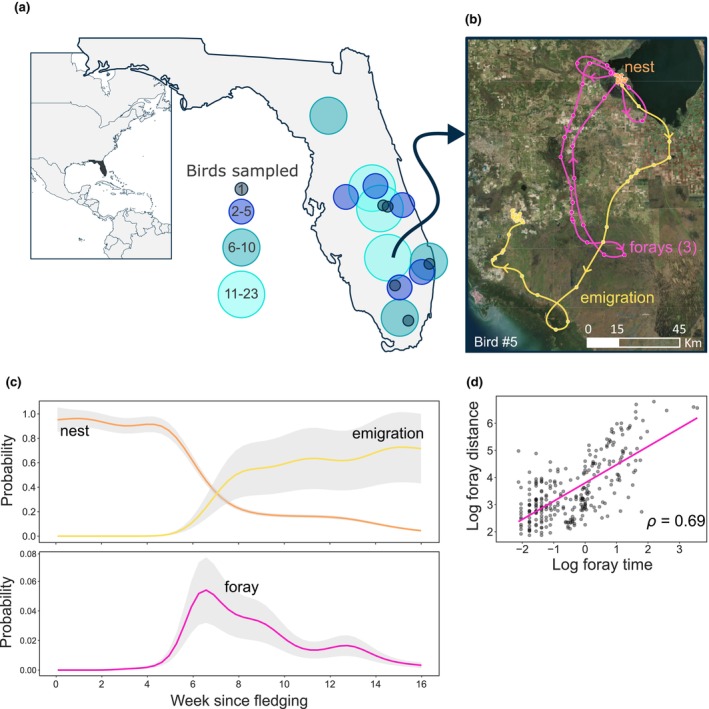
Study location and basic movement patterns of snail kites tagged with GPS‐GSM tags at fledging and tracked until emigration from the natal wetland. (a) Location of 16 wetlands (center of each circle) and the number of birds tagged in each, (b) a sample track from a single individual, showing hourly locations (circles) that begin at the nest, continue through forays, and end with emigration (arrows = direction of travel), (c) GAMM of the timing of nest, foray, and emigration for all birds combined (*n* = 104 tracked birds, estimate ±95% CI), and (d) the relationship between time spent in forays and distance traveled in forays (points = individual forays).

Our study area includes a network of 16 wetlands that are distributed within a 35,000 km^2^ area in Florida, USA, where snail kites breed (Figure 1). These wetlands consist primarily of palustrine habitat in the south, transitioning to lacustrine habitat in the north. Native and exotic apple snails occur within these wetlands and snail kites feed effectively on both species (Reichert et al., 2020). As part of an ongoing monitoring program, from 2000 to 2022 we located nests of snail kites and monitored them until fledging. These long‐term data were used to interpret the timing of nest initiation, the incubation/nestling period, and post‐fledging. To understand early exploratory movements, from 2016 to 2022 we deployed GPS tags on nestlings just prior to fledging.

Solar‐charging GPS‐GSM tags (Ecotone Telemetry, Cellular Tracking Technology, Ornitela) were used to monitor the movements of 104 juvenile snail kites (Figure 1a). The tags collected GPS locations that were accurate to ±10 m (C. Poli, unpublished), and transmitted data via cellular networks. Nestling birds that were close to fledging (23–31 days of age) were captured from nests by hand. Birds were selected opportunistically as fledglings became available, and we attempted to sample across a range of natal conditions (dry/wet), with replicate tagged individuals from each site and year. We did not sample more than one individual from any nest. Tags weighing 14–16 g (corresponding to 3%–5% of adult body mass; Barron et al., 2010) were attached to the back of individual birds using a wing harness. Tags were programmed to transmit hourly during daylight (~12 locations/day). Two feathers were taken from the breast or back and processed in a lab to determine sex of 70 of the tagged individuals (34 were not sexed due to logistical constraints), and birds were banded with USGS aluminum bands and metal bands with unique alpha‐numeric and color combinations.

### 2.2 Natal site characteristics

To understand hydrology at the natal site, we focused on hydrologic gauges maintained by natural resource agencies, which provide daily measures of the stage (defined as the height of the water surface above an established datum plane) at each wetland. Stage data have been used to successfully interpret nest survival in snail kites (Fletcher et al., 2021), and stage data are often used in wetland management decisions We extracted daily stage measures from gauges (DBHYDRO (Environmental Data), n.d.) at each natal wetland following Fletcher et al. (2021). For each bird, we calculated mean stage in the natal wetland during the first seven days post‐fledging (to match the timescale that is frequently used by decision‐makers to manage wetlands in this system, USFWS, 1999), and change in stage in the natal wetland during the first 30‐days post‐fledging (to match the approximate timescale of first movements out of wetlands). Since the time period of hydrologic relevance to movement is poorly known, we initially explored time scales of seven, 14, and 30 days post‐fledging but found that measures were correlated (*r* ≥ .6). Variation in elevation lead to broad differences in stage measurements among wetlands; therefore, we centered the stage data for each wetland on the average value across the time period, resulting in a measure of relative wetness/dryness based on stage.

For a subset of 32 snail kites, we sampled apple snail density near the nest during the first 2–4 weeks post‐fledging, a critical time for snail kite survival (Bennetts & Kitchens, 1999). At each focal nest, we measured snail density using a 1 m^2^ aluminum trap that was open on the top and bottom (Darby et al., 1999, 2012). Sampling was located in eight different wetlands and spanned 2016–2020, with 4–9 focal nests sampled per year. We threw the trap into the wetland in the vicinity of the nest with the open ends facing up/down and seated the trap into the substrate. We uprooted any vegetation contained within and searched the vegetation for snails. We then systematically swept the trap with a dip net and extracted all snails found in 12 sweeps. If a snail was found, it was counted and the sweeps started over at zero and continued until 12 sweeps were completed with no snails captured. We repeated this process multiple times, first in foraging habitat near the nest followed by additional throws located a random distance (range 10–100 m) and direction from the first throw. If a random throw was located within a shallow or densely vegetated area (i.e., unsuitable habitat for foraging; Darby et al., 2012), sampling was conducted in the nearest suitable habitat. Throws were repeated until either nine snails were captured in the first 10 throws (a high‐density site), zero or one snail was captured in 15 throws (a low‐density site), or 20 throws occurred (an intermediate‐density site; Darby et al., 2012). We estimated snail detection probability, *p*, (e.g., constant, overall detectability) by placing 1–3 marked snails into ~20% of the throw traps in each site (Darby et al., 1999). Dip net operators did not know if a marked snail was included. We divided the number of times dip net operators detected the marked snail by the number of times they failed to detect it to calculate *p*. At each nest *i*, we estimated snail density, $d_{i}$ (snails/m^2^), based on the total number of snails counted, ${\hat{N}}_{i}$, adjusted for detection as:

$$d_{i}=\frac{1}{\sum_{t=1}^{T}A_{t}}\times \left(\frac{{\hat{N}}_{i}}{p}\right)$$

where $A_{t}$ is the area sampled for throw *t* (1 m^2^).

### 2.3 Analysis

To understand the timing of movement behavior, we determined whether a given bird was near the nest, in foray, or had emigrated from the natal site for each GPS location (Figure 1b). We defined a snail kite foray as a recursive movement in which a bird leaves the boundaries of the natal wetland, or remains in the natal wetland but reaches a Euclidean distance ≥3 km from the nest (the minimum distance recorded for forays that left the natal wetland; relevant when the natal wetland is large), similar to methods in Cox and Kesler (2012). We defined movement near the nest for each bird as locations in the natal wetland that were not identified as forays. We identified the emigration date as the date at which birds ceased regular attendance of the nest area and did not return for at least 30 days (Bennetts & Kitchens, 2000). Since forays occurred rapidly compared to emigration (days versus weeks away from the natal site), it is unlikely that any events classified as emigration were actually forays where the individual suffered mortality partway through. To examine the timing of nest, foray, and emigration behavior, we used three generalized additive mixed models (GAMM; one for each behavior) with thin‐plate regression splines (since behaviors are non‐periodic), the default number of knots, and a Poisson distribution with a log link. The models included a binary (1/0) variable for the occurrence of a focal behavior at each GPS point as the response variable, week since fledging as the explanatory variable, and a random intercept of individual (Figure 1c).

For each bird, we calculated the time and total distance of each foray (Figure 1d). We then calculated two measures that describe initiation of forays: a binary variable for whether or not the bird forayed (1 = forays, 0 = no forays; hereafter foray behavior), and a continuous variable for time to first foray. We calculated three measures that describe activity during forays: number of forays, maximum distance from the nest attained during a foray, and total cumulative duration of a foray. Finally, we calculated a measure of departure from the natal site: the total time from fledging to emigration.

To determine the effects of hydrology on movement behavior, we used generalized linear mixed models (GLMM) with six movement measures as response variables (tested one at a time). For each movement measure, we explored several biologically relevant hydrologic relationships and compared 22 models that represented single, additive, and two‐way interactions between the explanatory variables of stage, stage^2^, change in stage, and sex (for relationships among variables see Figure S1). We opted for a relatively large model set since the relationship between hydrology and movement is underexplored. Linear effects assume that a movement measure either increases or decreases with stage, while quadratic effects allow for the potential that movement is highest or lowest at a moderate stage (e.g., shorter forays at low and high stage), which is relevant for kites that may move differently in response to hydrologic extremes compared to average conditions. Interactions between stage and change in stage (Δstage) were considered because different movement responses are expected when water in a wetland is deep and changing rapidly compared to when water is shallow and changing rapidly. We tested for effects to sex on foray behavior to account for potential sex‐biased dispersal, and since female and male snail kites tend to differ in body size, which could influence locomotion ability. We considered additive and interactive effects of sex to allow for the possibility that males and females may have the same responses to hydrology (no effect), the same response but different propensities for movement (additive), or fundamentally different responses (interaction). We included a model with natal wetland area only and a model with fledge month only in the model set. We used a binomial distribution and logit link for the probability of foray, a Poisson distribution and log link for the number of forays, and a gamma distribution and log link for all other GLMM. Using the subset of birds with snail sampling data, we examined whether snail density influenced movement behavior. We ran models for each movement measure with snail density and snail density^2^ as explanatory variables (two models), and with snail density and snail density^2^ as additive effects in the top hydrology model for each movement measure (two models), and compared them to the top hydrology‐only model for each movement measure. Throughout, we used model selection to choose among competing hypotheses, and ranked models based on Akaike's information criterion adjusted for small sample size (AICc; Burnham & Anderson, 2002). We examined and reported any competing models within 2 AICc of the top model and found that relationships were either similar to the top model (e.g., the direction of the regression coefficient did not vary across models for a give a parameter) or uninformative due to 95% confidence intervals that overlapped zero. For parsimony, we discuss only the top model from each set.

To account for natal cohort effects on movement that are known to occur in kites (Valle et al., 2017), we included a random effect of birth year in all GLMM. We initially tested a random effect of natal wetland but found that it did not impact the deviance explained compared to a random effect of year, so it was not included. Time of fledging may be especially influenced by hydrology because dry conditions prevail early in the year while wet conditions tend to occur mid‐late year, thus we tested each top model as‐is, and with fledge month added as an explanatory variable. Models that did not include fledge month received more support than those that did, so fledge month was removed from the analysis. To further understand whether the time to emigration varied between forayers and non‐forayers, we re‐ran the top hydrology model for time to emigration as‐is, with an additive effect of foray behavior, and with an interactive effect of foray behavior. We used model selection to determine the most supported model.

We expected that timing of emigration and foray behavior would affect survival of kites. To estimate survival, we analyzed monthly encounter histories for 99 of the 104 GPS‐tracked snail kites (two birds had incomplete encounter histories, and three birds that did not emigrate before tag failure were eliminated from analysis). We used GPS locations of the tracked kites to create encounter histories for each bird across nine survey periods per year (six surveys conducted 3 weeks apart from March–June plus one survey each in October, January, and February). These nine surveys are based on time periods of standardized resight surveys conducted throughout the breeding range as part of a long‐term monitoring program (Fletcher et al., 2021). We then used band resights for GPS birds to impute detections when the GPS did not provide any. We examined whether the resights contributed novel encounter information and found 13 resights of 9 birds that occurred after each bird's tracking device stopped transmitting GPS locations. These extra resights occurred 3.0–19.2 months after the tags failed (mean ± SD = 8.4 ± 5.1 months) and resulted in complementary encounter info for ~10% of birds. We, therefore, used extensions of the Cormack‐Jolly‐Seber model (CJS; Cormack, 1964; Jolly, 1965), to estimate apparent monthly survival (*Phi*; hereafter survival) and detection probabilities (*p*). Survival is considered “apparent” because this approach does not distinguish between mortality and permanent emigration from our study sites. CJS models acknowledge the potential that if individuals are not resighted or if GPS transmitters stop transmitting, observed patterns could be due to mortality or due to sampling‐related issues (e.g., transmitter failure, imperfect detection of banded kites).

We allowed apparent survival to vary temporally and to differ between age classes (juvenile: <1 year; adult: >1 year). To examine whether foray behavior alone led to changes in survival, we tested for additive and interactive effects between age class and foray behavior (1 = forays, 0 = no forays; two models). All subsequent models contained an interactive effect between age class and time to emigration because juvenile survival is much lower than adult survival (Reichert et al., 2020). We focused primarily on juveniles because we expected time to emigration to impact survival of juvenile birds more than adults (Bennetts & Kitchens, 1999; Poli et al., 2022). We tested for a linear and a quadratic effect of time to emigration, with an additive effect of foray behavior and an interactive effect of foray behavior (to account for potential opposing survival trends between forayers and non‐forayers; four models). Quadratic effects allow for the potential that survival is highest for birds that emigrate after a moderate amount of time as observed in Rotics et al. (2021). We compared these six models to null models of age class only and age class × time to emigration only. We tested variation in *p* by month, age class, and year. We found more support for an age class effect on detection than month or year, based on AICc, so we eliminated the month and year effects on *p* from our model set. Analyses were conducted in R 4.3 (R Core Team, 2023) and program MARK (White & Burnham, 1999).

## 3 RESULTS

The timing of movement behavior varied by individual, but all 104 tracked birds primarily remained near the nest site for the first 4 weeks post‐fledging, and 98.8% of observations were < 1 km from the nest during that time (Figure 1c). Eighty‐six individuals (82.7%) initiated forays and 89.6% of these forays crossed over a known wetland used for breeding other than the natal wetland. Forays tended to occur at 42.8 ± 11.5 (mean ± SD) days post‐fledging (range for time to first foray 20.3–93.3 days). These individuals made 3.3 ± 2.3 forays each (range for number of forays 1–9), which reached maximum distances of 49.9 ± 54.3 km from the nest (range for max foray distance 3.8–234.3 km). Each foray lasted 1.5 ± 1.8 days (range for mean foray duration 0.1–12.5 days). Emigration was initiated 56.0 ± 27.4 days after fledging (range for time to emigration 30.6–271.5 days). Three individuals (2.8%) either never emigrated, or tags failed without capturing emigration.

**FIGURE 2:**
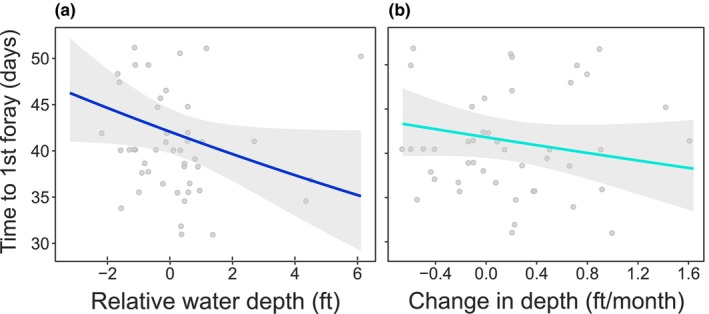
Behavior leading to forays is influenced by (a) natal hydrology (mean relative water depth (stage) during the first 7 days post‐fledging, and (b) change in water depth during the first 30 days post‐fledging; GLMM; Table 2). Results are from the most supported models ±95% prediction intervals. See Figure S2 for stage × ∆stage interaction.

Snail kites initiated forays earlier at higher stages and with rising water levels, based on the most supported model (Table 2, Figure 2; see Table S1 for the complete model set). Birds that experienced the highest stage in the week after fledging initiated forays ≤11.2 days earlier than those that experienced the lowest stage, according to predictions from the most supported model. Additionally, birds exposed to the highest rates of water ascension initiated forays ≤5.1 days earlier than those exposed to the highest rates of recession. None of the models considered for the probability of foraying had an effect that was clearly positive or negative (i.e., the 95% confidence intervals around β estimates broadly overlapped zero; Table 2). Effects were positive for the timing of foray initiation (Time to first foray *β*_stage × ∆stage_ = 0.03, 95% CI = 0.006, 0.07) and birds exposed to high stage and receding water forayed earlier than birds exposed to high stage and ascending water (Figure S2). In the subset of wetlands where we measured snail density, there was a weak tendency for forays to be further at higher snail density (Maximum foray distance: β = 0.4, 95% CI = −0.07–0.87; Tables S2 and S3; Figure S3). However, for the remaining movement measures, stage and ∆stage were more influential, or explained a similar amount of variance as the univariate snail density model, according to model selection (see Table S3 for all β estimates and 95% CIs for snail density).

**TABLE 2:** Model selection results of GLMM explaining the effects of hydrologic stage on six different movement measures (probability of foray, time to first foray, number of forays, mean foray duration, max foray distance, and time to emigration) for snail kites tracked with GPS from fledging through emigration in Florida, USA 2016–2022.

Probability of foray (*n* = 70 birds)	df	Deviance	AIC_c_	ΔAIC_c_	*w*_*i*_
Stage × ∆stage_**(+)**_	5	64.0	75.0	0.0	0.14
Stage_**(+)**_	3	69.0	75.5	0.5	0.11
Fledge month_**(−)**_	3	69.2	75.6	0.6	0.10
Sex_**(−)**_	3	69.6	76.0	1.1	0.08
STAGE_**(+)**_ + ∆stage_**(−)**_	4	67.8	76.4	1.4	0.07
Natal wetland area_**(−)**_	3	70.0	76.4	1.4	0.07
Stage × ∆stage_**(+)**_ + sex_**(−)**_	6	63.2	76.5	1.5	0.07
Stage_**(+)**_ + sex_**(−)**_	4	68.2	76.8	1.9	0.05
**Stage** × **∆stage**_**(+)**_ + stage^2^_(+)_ × ∆stage_(+)_	7	61.0	76.9	1.9	0.05
Stage_**(+)**_ + stage^2^_**(+)**_	4	68.2	76.9	1.9	0.05
Time to first foray (*n* = 55^a^)					
**Stage** × **∆stage**_**(−)**_	6	392.0	405.7	0.0	0.44
Number of forays (*n* = 56)					
Stage_**(+)**_ + stage^2^_**(+)**_ + **sex**_**(−)**_	5	230.4	241.5	0.0	0.18
**Stage**_**(+)**_ + **sex**_**(−)**_	4	232.8	241.5	0.0	0.18
Stage × **sex**_**(−)**_	5	230.8	242.0	0.5	0.14
**Stage** × ∆stage_**(−)**_ + sex_**(−)**_	6	229.4	243.2	1.7	0.08
**Stage**_**(+)**_ + ∆stage_**(−)**_ + **sex**_**(−)**_	5	232.2	243.5	2.0	0.07
Mean foray duration (*n* = 56)					
**Stage** × **sex**_**(−)**_	6	136.2	149.9	0.0	0.30
**Stage**_**(−)**_	4	142.4	151.2	1.2	0.16
Max foray distance (*n* = 56)					
**Stage** × **sex**_**(−)**_	6	531.0	544.8	0.0	0.32
Stage_**(−)**_	4	538.0	546.7	2.0	0.12
Time to emigration (*n* = 68)					
**∆stage**_**(−)**_	4	587.6	596.2	0.0	0.28
Stage_**(−)**_ + **∆stage**_**(−)**_	5	586.2	597.2	1.1	0.16
**∆stage**_**(−)**_ + sex_**(−)**_	5	586.8	597.9	1.7	0.12
**∆stage** × sex_**(−)**_	6	584.6	598.0	1.8	0.11

*Note*: Only models ≤2 AICc from the top model are shown, though 22 models were tested for each movement measure. Subscript (+) and (−) denote the direction of the β estimate for each parameter. Parameters for each model where the 95% confidence interval around β does not overlap 0 are in bold.

^a^ One outlier removed.

**FIGURE 3:**
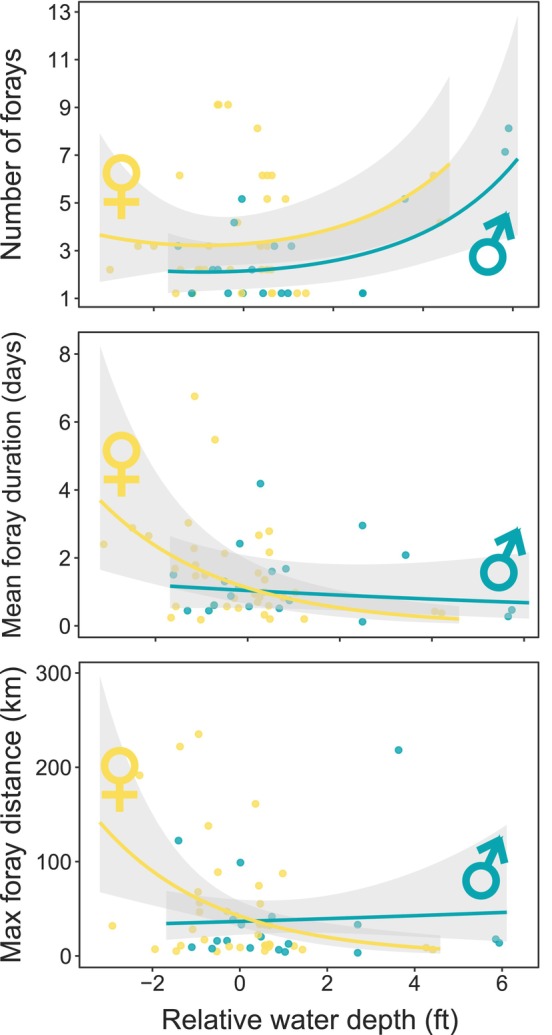
Behavior during forays is influenced by natal hydrology (mean relative water depth (stage) in the natal wetland during the first 7 days post‐fledging) and sex (GLMM; Table 2). Results are from the most supported models ±95% prediction intervals.

Females were more sensitive to natal hydrology than males, and differences in behavior during forays were most evident during the highest and lowest stages (Figure 3) according to the most supported models (Table 2). For both sexes, the number of forays increased with stage; however, females made 1.1–2.3 more forays than males that experienced the same post‐fledging water levels in the natal wetland. Males responded weakly to water levels, and those exposed to the highest stages made forays that were 0.49 days faster on average but 11.9 km farther from the nest (Euclidean distance of the longest foray) compared to those exposed to the driest conditions. Female movement behaviors, however, were strongly correlated with hydrology: they spent 3.5 days longer per foray and reached a maximum foray distance that was 134.3 km farther during the lowest stages compared to the highest stages, according to predictions from the most supported model (Figure 3).

**FIGURE 4:**
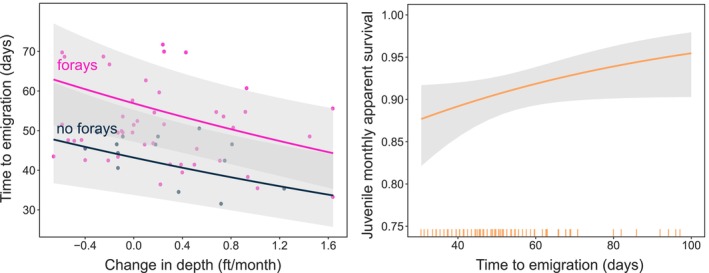
Emigration behavior is influenced by natal hydrology and differs depending on whether or not birds foray (GLMM; left panel), and monthly apparent survival of juvenile birds (≤1 year old) is highest for those that emigrate later from the natal wetland (right panel), according to the most supported models (Table 3) ± 95% prediction intervals.

The outcome of forays for emigration dates was mediated by hydrology, and the model that contained additive effects of ∆stage and foray behavior (df = 5, AICc = 591.5, *w*_*i*_ = 0.71) received more support than a model with an interaction between ∆stage and foray behavior (df = 6, AICc = 593.7, *w*_*i*_ = 0.22) or a ∆stage‐only model (df = 4, AICc = 596.2, *w*_*i*_ = 0.06). Birds that experienced relatively stable or slightly receding post‐fledging water levels took longer to emigrate compared to those that experienced ascending water (*β*_∆stage_ = −0.15, CI = −0.25 to −0.06). Across all rates of hydrologic change, snail kites that forayed also emigrated 10.7–15.1 days later than those that did not foray (*β*_forayers_ = 0.28, CI = 0.08–0.47), according to predictions from the most supported model (Figure 4).

Snail kites that emigrated later had higher monthly survival post‐emigration, regardless of whether they forayed (Figures 4 and S4), according to the most supported model (Table 3). Survival was highest for those that emigrated earliest (predictions at 30 days post‐fledging: *S* = 0.88, CI = 0.82–0.92), and lowest for those that emigrated latest (predictions at 100 days post‐fledging: *S* = 0.95, CI = 0.9–0.98). Compounded over 6 months, the survival rate for individuals that emigrate early (30 days post‐fledging) was 0.46 compared to 0.74 for individuals that emigrate late (100 days post‐fledging). In the second most supported model, survival of forayers was slightly higher than non‐forayers (*β*_forayers_ = 0.39, CI = −0.25 to 1.08), but the confidence interval around the parameter estimate for foraying overlapped zero. Detection (*p*) ranged from 0.88 (CI = 0.84–0.92) for juveniles to 0.96 (CI = 0.93–0.98) for adults.

**TABLE 3:** Model selection results explaining the effects of foray behavior and time to emigration on monthly apparent survival for 99 snail kites fledged from 16 wetlands in Florida, USA 2016–2022.

Model	Number of parameters	Deviance	AIC_c_	Δ AIC_c_	*w*_*i*_
**Age class × time to emigration**_**(+)**_	6	727.2	739.3	0.0	0.47
**Age class × time to emigration**_**(+)**_ + foray behavior_(+)_	7	725.9	740.0	0.7	0.34
Age class × time to emigration_**(+)**_ + age class × time to emigration^2^_**(+)**_ + foray behavior_**(+)**_	9	725.3	743.5	4.2	0.06
**Age class**_**(−)**_ + foray behavior_**(+)**_	5	668.0	744.1	4.8	0.04
**Age class**_**(−)**_ (null model)	4	670.3	744.3	5.0	0.04
Age class × time to emigration × foray behavior_**(+)**_	10	725.1	745.5	6.1	0.02
Age class × foray behavior_**(+)**_	6	667.5	745.6	6.3	0.02
Age class × time to emigration × foray behavior_**(+)**_ + **time to emigration** ^**2**^ **× foray behavior**_**(−)**_	12	723.3	747.7	8.4	0.01

*Note*: For all models, *p* = age class. Subscript (+) and (−) denote the direction of the β estimate for each parameter. Parameters for each model where the 95% confidence interval around β does not overlap 0 are in bold.

## 4 DISCUSSION

We found that early‐fledging habitat explained the initiation, execution, and outcome of foray behavior and that a consequence of foraying, delayed emigration, was linked to early‐life survival benefits. These results emphasize the fundamental role of natal habitat for determining important behavioral patterns, strengthen links between individual movement decisions and their demographic consequences, and provide a baseline for management of wetland systems for an endangered species during an important life stage.

### 4.1 Effects of the early environment on movement

Snail kite movement was related to water levels and change in water levels at the natal site following similar trends previously identified for reproduction and breeding dispersal (Robertson et al., 2017). Specifically, thresholds that lead to low nest survival occur at unusually high and low water levels and during high rates of hydrologic change (i.e., high ascension and recession rates; Fletcher et al., 2021). High water levels and high ascension rates may cause inundation and collapse of vegetation used for perching (esp. cattail/*Typha* sp.) and reduced snail availability in the rising water column (Bennetts et al., 2002; Darby et al., 2002; Sykes, 1987). Kites in this study moved earlier, forayed more times, and emigrated earlier under high and ascending conditions, which is consistent with movement behavior of animals in poor habitat (Stamps, 2006). At the opposite extreme, dry conditions and high recession rates in the study system correspond to reduced snail availability, vegetation community turnover, and increased predation risk, which also creates poor foraging habitat for kites (Darby et al., 2002; Robertson et al., 2017; Sykes, 1987). For birds that experienced relatively dry conditions, foray initiation was delayed and individuals made fewer trips. Given that kites avoided returning to poor natal habitat, foray behavior may be common simply because exploring birds fail to encounter better habitat than their home habitat, representing unsuccessful emigration attempts. Importantly, the driest natal conditions are expected to have negligible impacts fledgling movements because few nestlings survive (Beissinger & Snyder, 2002; Fletcher et al., 2021), but in less severe environments, it might be that hydrology has greater effects on fledglings than nest initiation and nest survival. Targeted tracking of animals that fledge in extreme conditions could help solidify the observed relationships.

The observed movement response to snail density was not consistent with hypotheses regarding prey abundance and exploration in snail kites. Kites are thought to explore when food is abundant, move little when food is moderate, and disperse when food is limited (Bennetts & Kitchens, 2000). In this study, snail kites that experienced higher snail densities at the natal site forayed slightly longer and farther than those that experienced lower snail densities. However, these results should be interpreted cautiously because of the limited number of sites that were sampled for snails and the small hydrologic range over which sampling occurred (Figure S3). Our sampling was likely limited to wetlands where snail density was relatively high, since it was paired with successfully fledged nests (see Cattau et al., 2014 for specific native snail densities associated with successful reproduction). Additionally, snail sampling was conducted near each nest, but snails may be patchily distributed within wetlands, and juvenile birds may forage away from the nest, leading to a potential mismatch between movement behavior and snail density as measured here.

The occurrence of forays in both sexes of snail kites in response to natal hydrology suggests that resource acquisition necessitates movement for all individuals (Reichert et al., 2016). In this system, both males and females must be prepared to relocate in response to regular but infrequent droughts (Martin et al., 2006; Takekawa & Beissinger, 1989). However, females were more sensitive to hydrology, especially at extremely high and low stage, which may indicate that there is local competition driven by changes in habitat and prey. Although interference competition does not commonly occur in this species (Reichert et al., 2020), existing differences in body size between males and females could allow for exploitative competition, which would favor larger, heavier females (Reichert et al., 2020). Individual condition can also drive aspects of dispersal including emigration timing (Robles et al., 2022), but in during analysis of the data presented here we found no effect of condition at fledging on snail kite movement metrics (C. Poli, unpublished). Inbreeding avoidance might also be important for exploring kites, since it is a major cause of sex‐biased dispersal (Pusey, 1987). Consistent with knowledge for inbreeding avoidance in birds, female snail kites forayed farther and longer than males, and prior research found that males tend to be more philopatric (Robertson et al., 2017). Finally, females may be accounting for spatial autocorrelation in patch quality by moving far when conditions in natal habitat are poor. Despite sex‐based differences in travel immediately post‐fledging, sex does not appear to impact overall movement rates or rangewide connectivity in this population at long time scales (Bowling et al., 2012; Reichert et al., 2016).

### 4.2 Foraying and the relationship to delayed emigration

Although an animal does not necessarily have to delay dispersal to benefit from foraying, we found that foraying was related to delays in emigration. Delayed emigration may allow forayers to gain information about the landscape, then recoup energy invested in exploration. For instance, in cooperative breeders, individuals do not disperse directly but instead use foraying as a strategy to locate high quality breeding habitat while accessing food, shelter, and information about predators within the natal territory (Koenig et al., 1992). By delaying, kites benefit from familiarity with the natal site and support from parents that may provide supplemental food up to 39–53 days after fledging (Reichert et al., 2020). Additionally, foraying could be a condition‐dependent behavior, such that the highest‐condition individuals are better able to bear the energetic costs of exploration (Long et al., 2021). Ultimately, the link between foraying and emigration timing suggests that the natal site is important to fledgling kites long after they appear to be independent.

### 4.3 Delayed emigration is linked to higher survival

Delays can sometimes be beneficial and sometimes detrimental for demography. Delayed dispersal has the potential to negatively impact individuals when dispersal is both required and synchronous (e.g., Fattebert et al., 2019; Robles et al., 2022). In this study, we observed benefits to delay, where birds that forayed emigrated at an older age (compared to non‐forayers), which led to higher survival rates. The importance of age for survival is well‐studied (Bennetts & Kitchens, 1999; Cox et al., 2014; Sergio et al., 2019), and it is expected that the impact of foraying would be secondary or indirect. Even so, foraying individuals may incur higher fitness than their non‐foraying counterparts, despite delays in reproduction (Kingma et al., 2016; Larsen & Boutin, 1994; Walters et al., 1992). Good natal habitat may, therefore, be key to informed dispersal, and young birds that are forced to depart early from poor or deteriorating habitat could be at a fitness disadvantage compared to those that are able to remain in good habitat. For kites that seek refuge from dry wetland conditions (e.g., drought or managed dry‐downs), a well‐developed understanding of the landscape may be critical to movement, survival, and successful reproduction in relatively poor years. Importantly, foraying is predicted to provide the largest advantage in heterogeneous landscapes (Conradt et al., 2003), which applies to snail kites that must cross over inhospitable forest, farmland, and urban habitat, to disperse between isolated wetlands. Although the binary (yes/no) measure of foraying examined here was not sufficient for interpreting survival directly, other aspects of foray behavior (e.g., timing, distance) or factors associated with late emigration but not foraying could affect survival and other fitness components.

### 4.4 The relationship between forays and dispersal

We found that foraying in a non‐cooperative bird was linked to a component of dispersal: emigration timing. Forays can be considered a component of dispersal, but they are also considered a general type of search strategy, since searching can happen for a range of reasons that are not necessarily dispersal‐related (e.g., Deuel et al., 2017; O'brien et al., 2014), and animals do not always depart even though they foray. In young animals, the foray period can be prolonged compared to the onset of permanent departure (~1.3 years Barve et al., 2020) such that separating the two processes can reveal previously unknown biological complexity. The biological motivations for forays and dispersal also differ in that the former is used primarily to gather information, while the latter is related to reproduction, but they are linked in several ways. Memory of the landscape that accumulates during exploration may allow animals to disperse efficiently (Lima & Zollner, 1996; Vuilleumier & Perrin, 2006), and the distance and direction of exploration often corresponds to the dispersal location (Cox & Kesler, 2012; Haughland & Larsen, 2004; but see Selonen & Hanski, 2010). Individual exploration patterns and dispersal may also be linked through behavioral syndromes, where an animal's phenotype consists of a suite of correlated traits (Sih et al., 2004). In this case, bold, proactive, aggressive individuals, may explore and disperse longer distances than their shy, reactive, less‐aggressive counterparts (Dingemanse et al., 2003). The broad and bimodally distributed foray distances observed in this study (Figure S1), coupled with movement strategies that differ by natal cohort in adult snail kites (Valle et al., 2017) may reflect behavioral syndromes related to movement in this species.

Movement ecology has long been concerned with linking fine‐scale behavior to fitness consequences, but studies that do so are uncommon, in part because it is difficult to estimate the mortality risk that might come with a short‐term movement decision (Fletcher Jr. et al., 2019). Population biology and metapopulation ecology have provided insight (usually at mesoscales, such as Robertson et al., 2017), and theoretical work outlines the consequences of foraying for dispersal (Conradt et al., 2003), yet information gaps in natural systems persist. This study is novel in that it not only examines environmental controls on fine‐scale movement, but goes a step farther to test for demographic impacts. Further empirical study is needed to understand the ways in which exploration might improve individual resilience to poor conditions over long timescales. Given accelerating rates of habitat loss, and the importance of movement for connecting habitat, attention should especially focus on the role of forays in highly fragmented landscapes.

### 4.5 Implications

These results have implications for conservation and management, particularly for species that live in unpredictable and variable systems. Broadly, exploration patterns can provide insight into colonization dynamics (Bowler & Benton, 2005) and age structure within populations (Holmes et al., 1996). A mechanistic understanding of the influence of environmental change on post‐fledging movement could be used to encourage recruitment to target areas or increase survival. Through manipulation of natal habitat, managers may have more control than they think over movement phenotypes and, therefore, life histories of important species.

**FIGURE 5:**
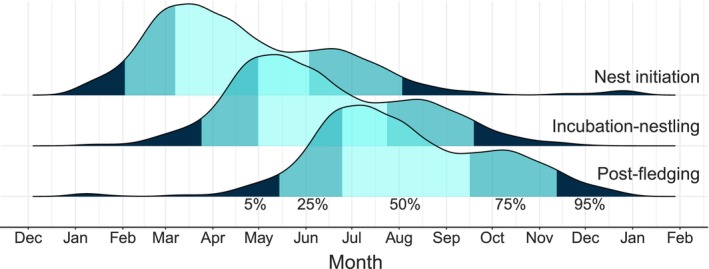
Timeline of extended nest management. Density plots indicate the timing of nest initiation (back‐calculated from observed hatching and fledging dates; top), a 56‐day period from incubation‐fledging (middle), and a 56‐day post‐fledging period (mean time to emigration in this study; bottom). Data shown are from 6267 snail kite nests monitored in Florida, USA, 2000–2022. Dark blue = 5th and 95th percentiles, medium blue = 25th and 75th percentiles, light turquoise = 50th percentile.

Current habitat management for snail kites focuses heavily on the period from nest initiation through fledging. Less attention has been paid to the post‐fledging period, in part because it occurs later in the year than nesting and the factors that control juvenile survival are complex. However, this research ties foray and emigration behaviors to conditions at the natal site, supporting the need for extended nest‐site management (Figure 5) that includes the period between fledging and emigration. Hydrologic targets for post‐fledging are likely to differ from targets for nesting because of an annual wet cycle of high rain and flooding that begins late in the breeding season. When combined with existing knowledge of reproduction, hydrologic models have the potential to guide management of wetland systems throughout the snail kite annual cycle (Fletcher et al., 2021), which is critical to increasing and sustaining the U.S. population of this highly endangered species. These results also emphasize the importance of maintaining a network of discrete wetlands that give fledglings options for habitat during the full calendar year. Successful implementation may require consideration of tradeoffs given that these wetlands must accommodate a suite of species and stakeholders and that annual dry and wet cycles further complicate hydrologic management. Notably, extended nest‐site management likely applies to other species where reproduction and early‐life survival are limiting, and may be particularly helpful for conserving long‐lived species.

This study has implications for interpreting movement trajectories, and especially emphasizes the necessity of considering movement at moderate scales. Forays would not be well captured with conventional segmentation and latent state methods (e.g., hidden Markov models), but they are important for dispersal and should not be ignored. Movement analyses need to move effectively beyond step lengths and turning angles to recover large‐scale variation in systematic search behaviors such as forays. Doing so can reveal the importance of movement for not only individuals but also populations, which is a major goal in ecology (Nathan et al., 2022).

## AUTHOR CONTRIBUTIONS

**Caroline L. Poli:** Conceptualization (lead); data curation (lead); formal analysis (lead); funding acquisition (equal); investigation (lead); methodology (equal); project administration (supporting); resources (equal); software (lead); supervision (supporting); validation (equal); visualization (lead); writing – original draft (lead); writing – review and editing (lead). **Kenneth D. Meyer:** Conceptualization (equal); funding acquisition (equal); investigation (supporting); methodology (supporting); project administration (supporting); resources (supporting); supervision (supporting); writing – review and editing (supporting). **Philip C. Darby:** Conceptualization (equal); funding acquisition (equal); methodology (supporting); project administration (supporting); resources (supporting); supervision (supporting); writing – review and editing (supporting). **Sarah J. Dudek:** Data curation (supporting); formal analysis (supporting); investigation (supporting); writing – review and editing (supporting). **Gina Kent:** Data curation (supporting); investigation (supporting); methodology (supporting); resources (supporting). **Robert J. Fletcher Jr.:** Conceptualization (lead); data curation (equal); formal analysis (supporting); funding acquisition (lead); investigation (supporting); methodology (equal); project administration (lead); resources (lead); software (supporting); supervision (lead); validation (supporting); visualization (supporting); writing – review and editing (equal).

## FUNDING INFORMATION

Funding was provided by US Army Corps of Engineers (W912HZ‐19‐2‐0037, W912HZ‐20‐2‐0033), Florida Fish and Wildlife Conservation Commission (13416 TA No 2223, 13416‐TA‐21A02), US Geological Survey (G16AC00273), South Florida Water Management District (9500009577), St John's River Water Management District (34941), and the Everglades Foundation (117140).

## CONFLICT OF INTEREST STATEMENT

The authors declare no conflicts of interest.

## Supporting information


Data S1.

## Data Availability

All datasets and code generated for this study are available in the Figshare repository (https://doi.org/10.6084/m9.figshare.25155626) by searching the title of the paper.
