# Supplementary material for: Foray movements are common and vary with natal habitat for a highly mobile bird
Source: Ecol Evol. 2024 Feb 29;14(3):e11096. doi: 10.1002/ece3.11096 (PMC10904963; doi:10.1002/ece3.11096)
Supplement: Supplementary file 1 — Data S1. [file ECE3-14-e11096-s001.docx]

Supporting Information for

**Foray movements are common and vary with hydrology in natal habitat for a highly mobile bird**

This Supporting Information includes:

a) Correlation matrix for response (movement measures) and explanatory variables (hydrology, snail density)

b) Complete models set and results from GLMM evaluating the effects of hydrology on movement

c) Model selection results and estimates for movement and snail density models

d) Plot of adult survival from the interaction between age class and time to emigration in the most supported survival model


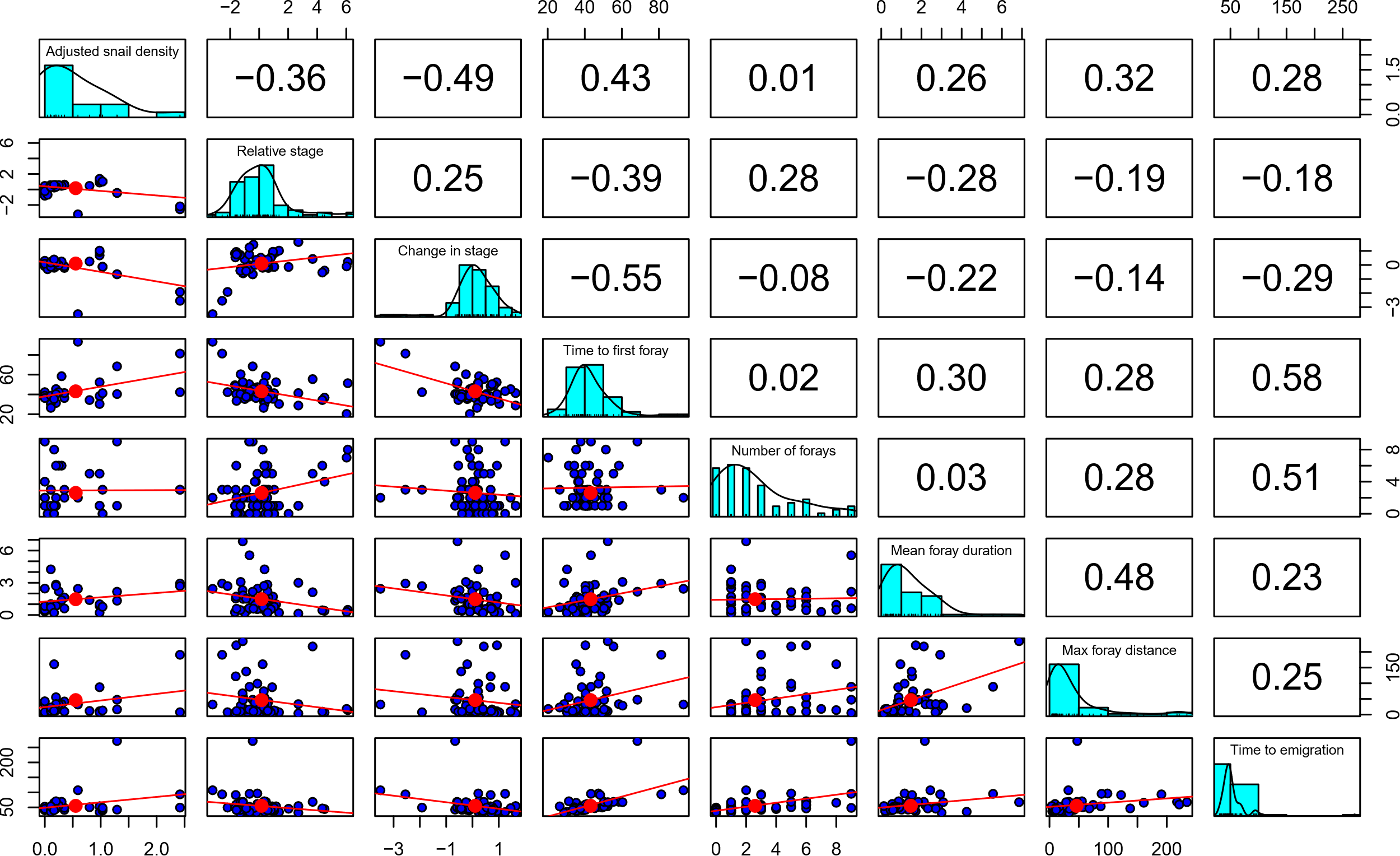
**a) Correlation matrix for response (movement measures) and explanatory variables (hydrology, snail density)**

**Figure S1.** Correlation matrix of six movement measures, hydrology, and snail density. Scatter plots in the lower off-diagonal show a linear regression fit (red lines) and center of the data (large red dot). The diagonal shows histograms and the upper off-diagonal shows the Pearson correlations.

**b) Complete models set and results from GLMM evaluating the effects of hydrology on movement**

**Table S1.** Full model set (22 models) and AICc values from GLMM explaining the effects of hydrologic stage on six different movement measures (probability of foray, time to first foray, number of forays, mean foray duration, max foray distance, and time to emigration) for snail kites tracked with GPS from fledging through emigration in Florida, USA 2016–2022.

| **Model** | **Probability of foray** | **Number of forays** | **Time to first foray** | **Mean foray duration** | **Max foray distance** | **Time to emigration** |
| --- | --- | --- | --- | --- | --- | --- |
| sex | 76.0 | 426.1 | 250.1 | 148.6 | 548.9 | 604.5 |
| natal wetland area | 76.4 | 426.8 | 252.2 | NA | NA | 604.7 |
| fledge month | 75.6 | 426.4 | 252.0 | 146.7 | 546.8 | 603.1 |
| stage | 75.5 | 417.3 | 245.0 | 142.6 | 546.7 | 602.8 |
| stage + stage^2^ | 76.9 | 410.7 | 244.5 | 144.9 | 547.9 | 601.8 |
| stage + sex | 76.8 | 419.7 | 241.5 | 144.6 | 549.2 | 603.5 |
| stage × sex | 79.1 | 417.9 | 242.0 | 142.8 | 544.8 | 604.9 |
| stage + stage^2^ + sex | 78.4 | 413.2 | 241.5 | 147.1 | 550.4 | 603.4 |
| stage × sex + stage^2^ × sex | 83.0 | 416.7 | 244.1 | 145.0 | 550.0 | 606.3 |
| ∆stage | 78.3 | 409.6 | 263.2 | 142.9 | 548.5 | 596.2 |
| ∆stage + sex | 77.7 | 412.0 | 252.4 | 145.1 | 550.5 | 597.8 |
| ∆stage × sex | 79.8 | 411.6 | 253.0 | 143.7 | 552.5 | 597.9 |
| stage + ∆stage | 76.4 | 408.7 | 246.2 | 143.2 | 548.8 | 597.2 |
| stage + stage^2^ + ∆stage | 78.4 | 409.6 | 246.9 | 144.2 | 550.4 | 599.4 |
| stage × ∆stage | 75.0 | 405.7 | 244.0 | 143.5 | 551.3 | 599.4 |
| stage × ∆stage + stage^2^ × ∆stage | 76.9 | 410.0 | 246.5 | 139.5 | 549.2 | 602.7 |
| stage + ∆stage + sex | 78.1 | 411.1 | 243.5 | 144.8 | 551.4 | 599.2 |
| stage + stage^2^ + ∆stage + sex | 80.1 | 412.1 | 243.9 | 145.5 | 553.0 | 601.5 |
| stage × ∆stage + sex | 76.5 | 408.3 | 243.2 | 145.8 | 553.9 | 601.4 |
| stage × ∆stage + stage^2^ × ∆stage + sex | 78.9 | 412.8 | 245.2 | 142.1 | 551.5 | 604.7 |
| stage × sex + ∆stage × sex | 82.7 | 412.1 | 244.5 | 142.5 | 550.0 | 601.0 |
| stage × sex + stage2 × sex + ∆stage × sex | 87.5 | 417.6 | 248.9 | 143.3 | 555.7 | 602.8 |

**Figure S2.** Combined influence of mean relative water depth and change in depth (receding water= negative ∆stage, ascending water=positive ∆stage) on time to first foray (from GLMM; Table 2). Results are from the most supported model ± 95% prediction interval.


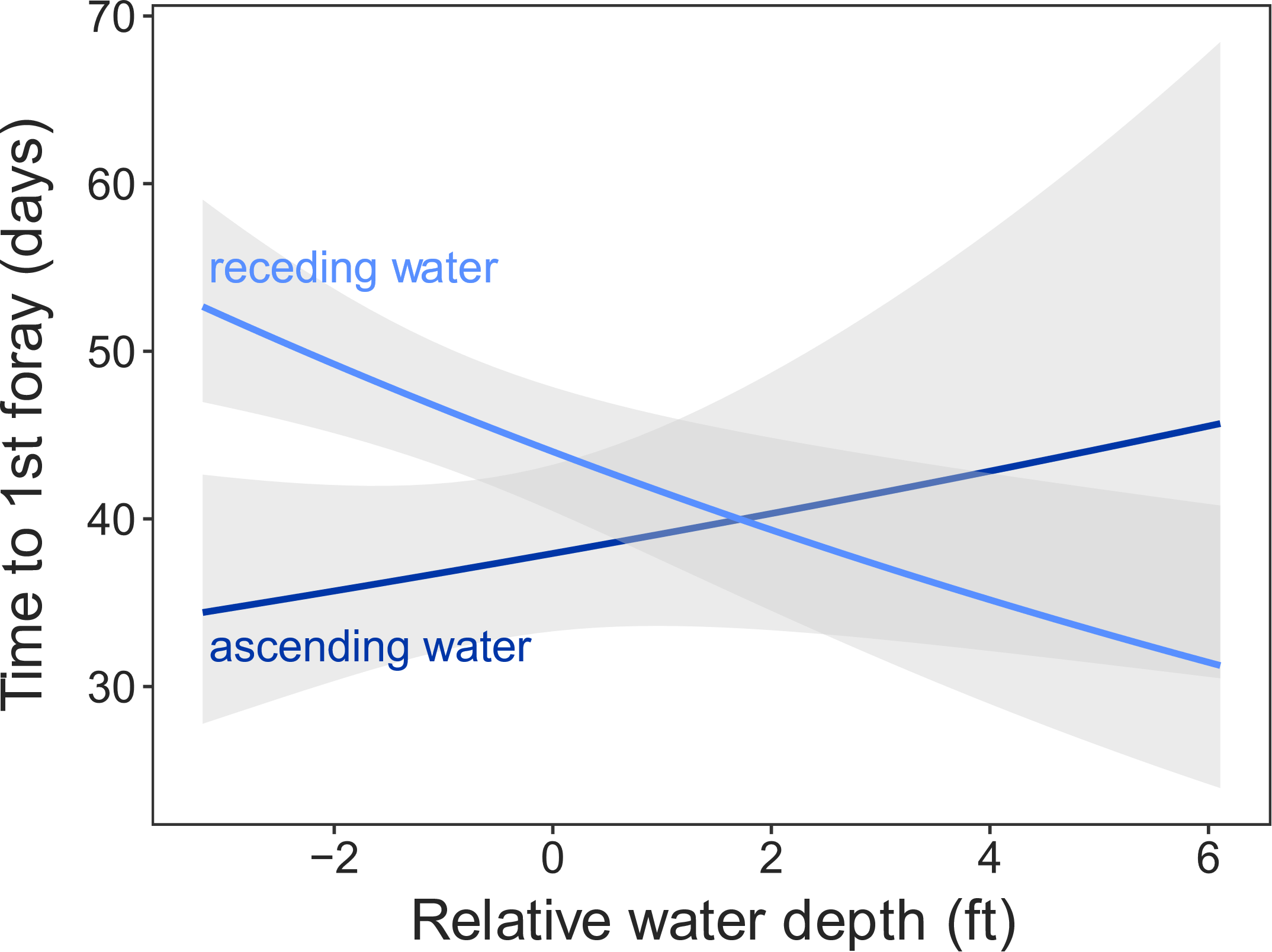


**c) Model selection results and estimates for movement and snail density models**

**Figure S3.** Frequency distribution of post-fledging hydrologic conditions in the natal wetland for all snail kites (gray; N=70), compared to the subset of birds with a natal site that was sampled for snails (blue; N=32).


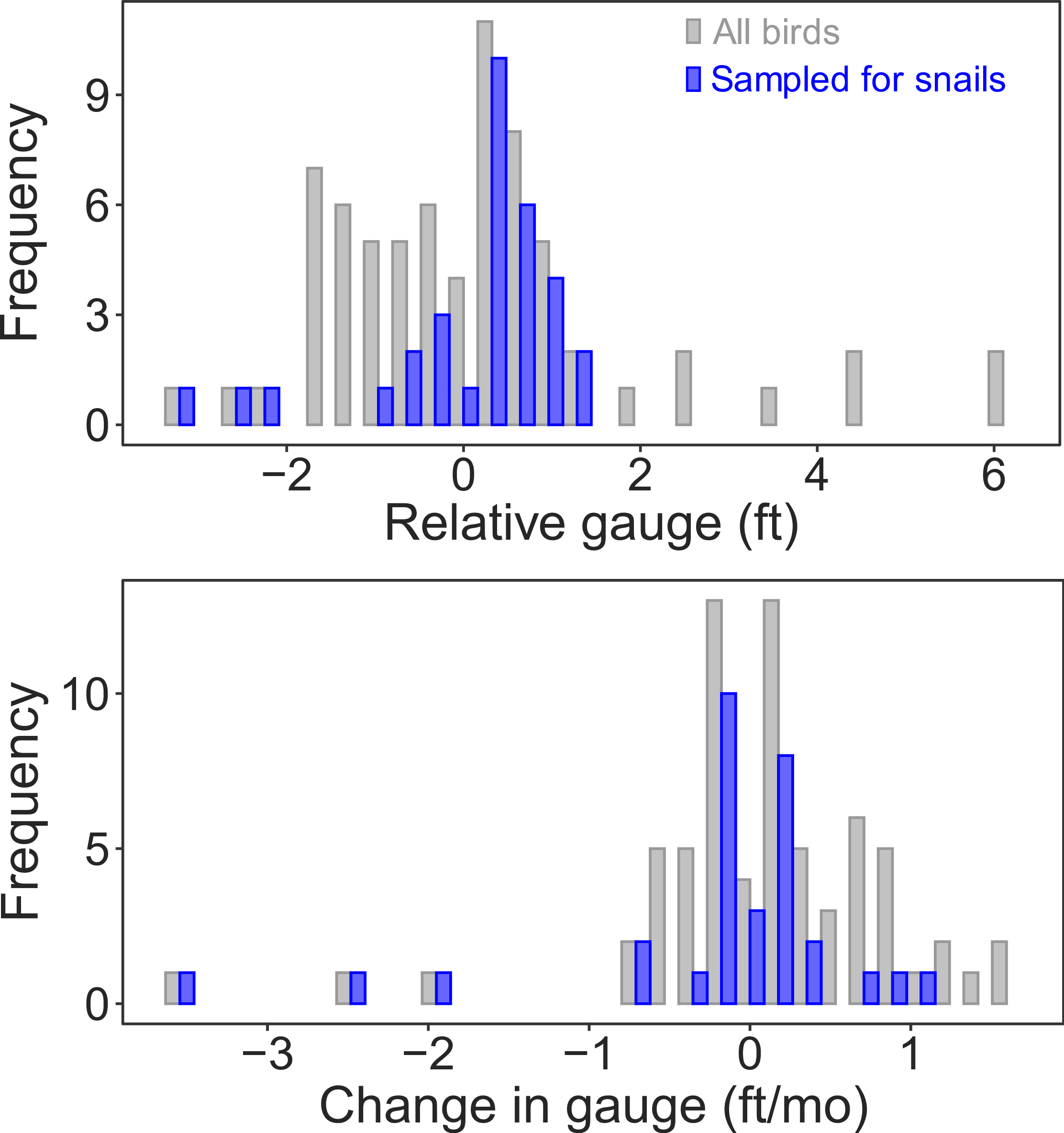


**Table S2.** Model selection results of GLMM explaining the effects of hydrologic stage and snail density on six different movement measures (probability of foray, time to first foray, number of forays, mean foray duration, max foray distance, and time to emigration) for snail kites tracked with GPS from fledging through emigration in Florida, USA 2016–2022. Models within 2 AIC of the top model are bold.

| **Probability of foray (n=32 birds)** | **df** | **AIC_c_** | **ΔAIC_c_** | **w_i_** |
| --- | --- | --- | --- | --- |
| **snail density** | **3** | **34.5** | **0** | **0.471** |
| **stage × ∆stage** | **5** | **35.7** | **1.23** | **0.254** |
| snail density + snail density^2^ | 4 | 36.6 | 2.14 | 0.162 |
| stage × ∆stage + snail density | 6 | 38.7 | 4.21 | 0.057 |
| stage × ∆stage + snail density + snail density^2^ | 6 | 38.8 | 4.26 | 0.056 |
| **Time to first foray (n=27 birds)** |  |  |  |  |
| **stage × ∆stage** | **6** | **205.9** | **0** | **0.786** |
| stage × ∆stage + snail density | 7 | 209.1 | 3.26 | 0.154 |
| stage × ∆stage + snail density + snail density^2^ | 8 | 211.2 | 5.29 | 0.056 |
| snail density | 4 | 216.4 | 10.58 | 0.004 |
| snail density + snail density^2^ | 5 | 219 | 13.17 | 0.001 |
| **Number of forays (n=27 birds)** |  |  |  |  |
| **stage + stage^2^ + sex** | **5** | **118.2** | **0** | **0.729** |
| stage + stage^2^ + sex + snail density | 6 | 121.3 | 3.02 | 0.161 |
| stage + stage^2^ + sex + snail density + snail density^2^ | 7 | 122.1 | 3.86 | 0.106 |
| snail density | 3 | 128.8 | 10.58 | 0.004 |
| snail density + snail density^2^ | 4 | 131.6 | 13.32 | 0.001 |
| **Mean foray duration (n=27 birds)** |  |  |  |  |
| **snail density** | **4** | **74** | **0** | **0.507** |
| **stage × sex** | **6** | **75.3** | **1.28** | **0.268** |
| snail density + snail density^2^ | 5 | 76.1 | 2.11 | 0.177 |
| stage × sex + snail density | 7 | 79 | 4.92 | 0.043 |
| stage × sex + snail density + snail density^2^ | 8 | 82.9 | 8.82 | 0.006 |
| **Max foray distance (n=27 birds)** |  |  |  |  |
| **snail density** | **4** | **253.6** | **0** | **0.675** |
| snail density + snail density^2^ | 5 | 255.8 | 2.15 | 0.23 |
| stage × sex | 6 | 257.9 | 4.3 | 0.079 |
| stage × sex + snail density | 7 | 261.4 | 7.78 | 0.014 |
| stage × sex + snail density + snail density^2^ | 8 | 265.1 | 11.51 | 0.002 |
| **Time to emigration (n=32 birds)** |  |  |  |  |
| **∆stage** | **4** | **237.2** | **0** | **0.736** |
| ∆stage + snail density | 5 | 239.7 | 2.47 | 0.214 |
| ∆stage + snail density + snail density^2^ | 6 | 242.6 | 5.39 | 0.05 |
| snail density | 4 | 254.6 | 17.32 | 0 |
| snail density + snail density^2^ | 5 | 256.8 | 19.61 | 0 |

**Table S3.** β estimates and 95% CIs for snail density from GLMM of six different movement measures and model selection (See Table S1 for model selection results). Note that for time to first foray, number of forays, and time to emigration, the most supported model did not include snail density. For probability of foray and mean foray duration, snail density and hydrology-only models performed similarly (were within 2 AICc). For max foray distance, snail density was the top model.

|  | **β (snail density)** | **LCL** | **UCL** |
| --- | --- | --- | --- |
| Probability of foray | 0.24 | -1.39 | 1.88 |
| Number of forays | 0.11 | -0.28 | 0.51 |
| Time to first foray | 0.04 | -0.09 | 0.18 |
| Mean foray duration | 0.21 | -0.17 | 0.60 |
| Max foray distance | 0.40 | -0.07 | 0.87 |
| Time to emigration | -0.04 | -0.16 | 0.08 |

**d) Plot of adult survival from the interaction between age class and time to emigration in the most supported survival model**

**Figure S4.** Monthly apparent survival of adult birds (>1 year old) from the most supported model (Table 3) ± 95% prediction intervals.

**
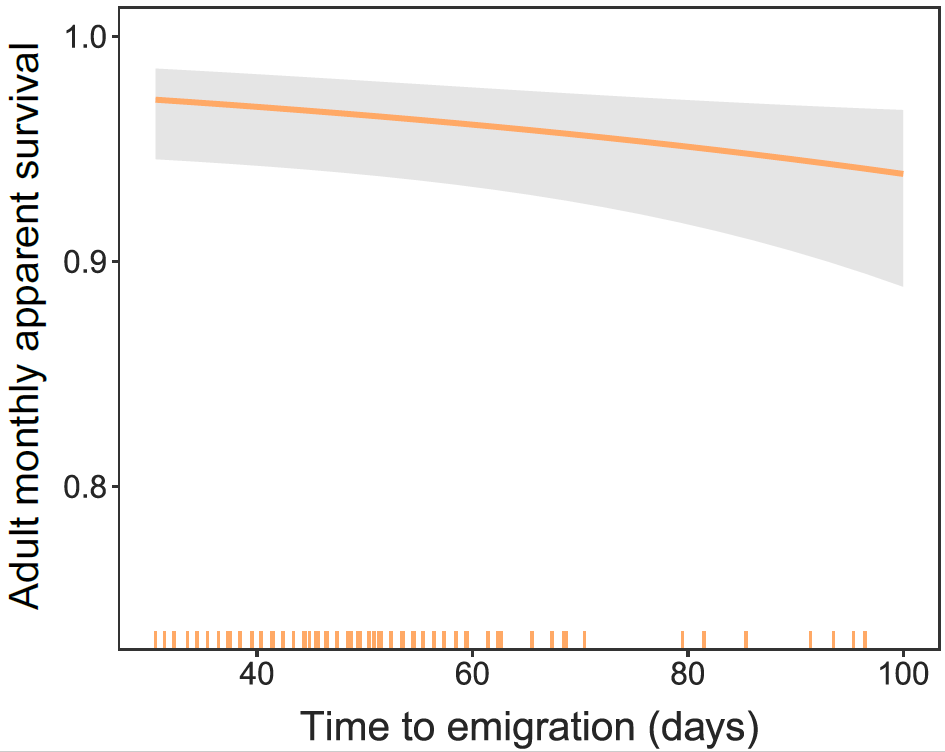
**
